# Assessing the Usability of a Novel Wearable Remote Patient Monitoring Device for the Early Detection of In-Hospital Patient Deterioration: Observational Study

**DOI:** 10.2196/36066

**Published:** 2022-06-09

**Authors:** Edward Itelman, Gadi Shlomai, Avshalom Leibowitz, Shiri Weinstein, Maya Yakir, Idan Tamir, Michal Sagiv, Aia Muhsen, Maxim Perelman, Daniella Kant, Eyal Zilber, Gad Segal

**Affiliations:** 1 Chaim Sheba Medical Center Sheba Beyond Virtual Hospital Ramat Gan Israel

**Keywords:** remote patient monitoring, noninvasive monitoring, general ward, early warning score system, patient deterioration, clinical prediction, wearable devices, uHealth

## Abstract

**Background:**

Patients admitted to general wards are inherently at risk of deterioration. Thus, tools that can provide early detection of deterioration may be lifesaving. Frequent remote patient monitoring (RPM) has the potential to allow such early detection, leading to a timely intervention by health care providers.

**Objective:**

This study aimed to assess the potential of a novel wearable RPM device to provide timely alerts in patients at high risk for deterioration.

**Methods:**

This prospective observational study was conducted in two general wards of a large tertiary medical center. Patients determined to be at high risk to deteriorate upon admission and assigned to a telemetry bed were included. On top of the standard monitoring equipment, a wearable monitor was attached to each patient, and monitoring was conducted in parallel. The data gathered by the wearable monitors were analyzed retrospectively, with the medical staff being blinded to them in real time. Several early warning scores of the risk for deterioration were used, all calculated from frequent data collected by the wearable RPM device: these included (1) the National Early Warning Score (NEWS), (2) Airway, Breathing, Circulation, Neurology, and Other (ABCNO) score, and (3) deterioration criteria defined by the clinical team as a “wish list” score. In all three systems, the risk scores were calculated every 5 minutes using the data frequently collected by the wearable RPM device. Data generated by the early warning scores were compared with those obtained from the clinical records of actual deterioration among these patients.

**Results:**

In total, 410 patients were recruited and 217 were included in the final analysis. The median age was 71 (IQR 62-78) years and 130 (59.9%) of them were male. Actual clinical deterioration occurred in 24 patients. The NEWS indicated high alert in 16 of these 24 (67%) patients, preceding actual clinical deterioration by 29 hours on average. The ABCNO score indicated high alert in 18 (75%) of these patients, preceding actual clinical deterioration by 38 hours on average. Early warning based on wish list scoring criteria was observed for all 24 patients 40 hours on average before clinical deterioration was detected by the medical staff. Importantly, early warning based on the wish list scoring criteria was also observed among all other patients who did not deteriorate.

**Conclusions:**

Frequent remote patient monitoring has the potential for early detection of a high risk to deteriorate among hospitalized patients, using both grouped signal-based scores and algorithm-based prediction. In this study, we show the ability to formulate scores for early warning by using RPM. Nevertheless, early warning scores compiled on the basis of these data failed to deliver reasonable specificity. Further efforts should be directed at improving the specificity and sensitivity of such tools.

**Trial Registration:**

ClinicalTrials.gov NCT04220359; https://clinicaltrials.gov/ct2/show/NCT04220359

## Introduction

Validated tools for the early identification of high risk for clinical deterioration in hospitalized patients, or early warning score (EWS) systems, would be of high medical value in clinical practice. A large meta-analysis of previous studies tried to evaluate the effectiveness of rapid response teams for the reduction of in-hospital death in such circumstances [1]. The analysis failed to reach firm conclusions owing to the low quality of design and subsequent outcomes of such studies. One potential cause could be the fact that in different clinical scenarios (eg, general wards vs surgical departments) the clinical circumstances, the classifications used to define the clinical deterioration, and the competencies of the clinical staff are heterogenous [2,3]. Therefore, ideally, early detection technologies and applied prediction algorithms should be tailored for specific patient populations and clinical scenarios.

A common method, used worldwide in general-internal medicine departments, for the early identification of deterioration is placing the patient in a telemetry bed. A retrospective analysis of the effectiveness and potential abuse of this method found that when analyzed retrospectively, only one-quarter of telemetry days during hospitalization were deemed appropriate [4]. Moreover, they described that eliminating unnecessary telemetry days would result in significant cost saving. Interestingly, they did not find any cases of deterioration among patients who were not connected to telemetry devices. This shows that the medical staff was highly professional yet too sensitive, having admitted patients to the telemetry bed frequently.

Possible ways to generate an early risk identification flag is to rely on automatically grouped physiological signals incorporated into different scoring systems, or using artificial intelligence (AI)-based computerized algorithms, rather than counting on follow-up observations by professional staff members. For example, a multicenter retrospective analysis of electronic health records’ data from all patients admitted to 5 US hospitals during the years 2008-2013 showed that prediction of the composite outcome of in-hospital cardiac arrest, the need for intensive care unit (ICU) transfer, and death within 24 hours of observation were higher when conducted using a computerized score [5]. This was also found in the setting of the high-acuity area of an emergency department [6]. Nevertheless, training and competency of professional staff members are key components in every program intended to assimilate computerized predictive tools in hospital departments [7].

Unlike the standard spot-check vital sign measurements that are conducted over a short period and could miss changes in parameters, frequent and automated vital sign collection for longer periods using remote patient monitoring (RPM) platforms with data transmission into algorithm-based computerized systems will potentially be better equipped to detect early changes and alerts of various risks [8-11]. It is also accepted that such systems would be of benefit if they are simple and easy to use, frequently measure multiple vital signs, incorporated into the workflow of the health care providers, improve patient outcomes, and would be of help to the medical staff in addition to other measures to improve patient surveillance [11-14]. We expect monitoring systems and early warning scores to be sensitive and specific. The currently used EWS systems, which are collected infrequently by the medical teams, are known to have a relatively high sensitivity and low specificity [15].

This study aimed to assess whether frequent RPM has the potential for early detection of the risk to deteriorate, using grouped signal-based scores, compared to clinical detection of deterioration by the medical staff.

## Methods

### Study Design and Overview

This prospective observational clinical study with retrospective analysis of the data was conducted in 2 general wards of a large tertiary medical center. Patients determined to be at high risk to deteriorate upon admission and assigned to a telemetry bed were included, after signing an informed consent form. On top of the standard telemetry overhead monitoring devices used in the general ward (Mindray; e PM 10M), a wireless, wearable monitor was attached to the chest of each patient, and monitoring was conducted in parallel. Multimedia Appendix 1 shows a CONSORT (Consolidated Standards of Reporting Trials) flowchart of the study.

Medical treatment was provided on the basis of standard monitoring system only, as the data gathered by the wearable monitor were analyzed retrospectively, with the medical staff being blinded to it in real time. The physiological data from the wearable monitors were collected automatically every 5 minutes during the first 72 hours from admission, with no personally identifiable information besides serial numbers of the devices. Inclusion criteria were adults (aged >18 years) transferred from the emergency department and admitted to the general wards, who were determined to be at an increased risk for clinical and physiological deterioration during the first 72 hours from admission by the attending physicians (eg, patients who were hemodynamically or respiratory unstable in the emergency department, patients suspected of arrhythmia or acute coronary syndrome, and those suspected with infection and signs of sepsis). Exclusion criteria were lack of informed consent, physicians' assessment that patients will not stay in the general ward for the entirety of the first 72 hours, and technical inability to attach the chest monitor to the patients. Furthermore, patients already defined as necessitating lifesaving procedures were not included in the study.

### Study Setting

Designated communication routers were deployed and installed in the departments to ensure continuous monitoring, data transmission, and automatic data collection of all measurements. Data were transferred through Bluetooth from the devices to the routers and through Wi-Fi from the routers to a data cloud. Data gathered during the first 72 hours post admission, including physiological parameters measured by the wearable monitors and clinical data and vital sign data collected using the standard-of-care devices and documented in the electronic medical record system, were retrospectively analyzed at the end of the collection phase.

### Early Warning of the Risk for Deterioration

Several EWSs of the risk for deterioration were used. These included (1) the National Early Warning Score (NEWS, described in Multimedia Appendix 2) [16-19]; (2) Airway, Breathing, Circulation, Neurology, and Other (ABCNO) score (described in Multimedia Appendix 3) [20]; and (3) deterioration criteria defined by the clinical team as what they expect to have from a device providing continuous monitoring (a “wish list” score, described in Multimedia Appendix 4). In all three scoring systems, the risk scores were calculated every 5 minutes using the frequent data collected by the wearable devices. Data generated by these early warning scores were compared with those obtained from the clinical records of actual deterioration among these patients. Actual clinical deterioration of patients was defined by the medical staff as (1) needing cardiopulmonary resuscitation, (2) needing to be transferred to the ICU, (3) dead or dying, or (4) deteriorating as defined by the ABCNO criteria, relying on measurements from currently used devices in the wards and without using data derived from the wearable monitors.

### The Wearable Monitoring Platform

Frequent monitoring was achieved using a wireless, noninvasive, wearable reflective photoplethysmography-based sensor (BB-613WP, Biobeat Technologies Ltd). The data were automatically transmitted immediately upon capture to a cloud-based web platform repository. Patients’ values recorded every 5 minutes included 13 physiological parameters, including heart rate, blood oxygen saturation, respiratory rate, cuffless blood pressure, stroke volume, cardiac output, cardiac index, systemic vascular resistance, heart rate variability, pulse pressure, mean arterial pressure, temperature, and single-channel electrocardiograms [21-25].

### Statistical and Data Analysis

We compared various EWS systems using the data collected via the continuous wearable monitoring system, with the exact time as recorded in the electronic medical record, where patients deteriorated, as detected by the medical teams.

Baseline physical parameters were calculated by averaging the first 12 measurements. Continuous data are expressed as mean (SD) values if normally distributed or median (IQR) values if skewed. Categorical variables are presented as frequency (%) values. Between-group comparisons of numerical values were carried out using an independent samples *t* test, followed by the Levene test for equality of variances. Chi-square and the Fisher exact test were used for between-group comparisons of categorial parameters. Early warning based on NEWS was defined as the initial time point in which the score was above 5. Early warning based on the ABCNO score and the local medical staff deterioration (“wish list”) criteria was defined as being detected when two consecutive measurements were above or below the defined thresholds (see Multimedia Appendices 3 and 4). Patients were included in the final analysis if more than 200 sessions of measurements (each session includes 13 physiological parameters and an EWS score) were carried out per patient, which was considered the minimal volume of data to analyze deterioration during the monitoring period. We did not treat any missing data; once patients had more than 200 measurement sessions, they were considered eligible for inclusion and further analysis.

The actual clinical deterioration events detected by the medical teams on site were collected, and the sensitivity and specificity of early warning by the wearable monitoring platform based on the 3 approaches—NEWS, ABCNO score, and “wish list” criteria of changes in parameters—were assessed post hoc, relying on the combination of the documented events in the electronic medical records of the patients and the physiological data collected by the wearable monitoring system. The investigators who made these post hoc assessments were blinded to the clinical outcomes of participating patients. Another element assessed was the warning time defined as the difference (in hours) from the early detection by any of these approaches to the actual clinical detection of deterioration as documented by the medical staff. All descriptive statistical analyses were performed using SPSS (version 25; IBM Corp).

Within the context of this study, readings regarded as “spurious readings” (basic definitions of either bad signals or signals defined as out of the sensor's measurement range) were automatically removed by the monitoring platform's algorithm and not included in the analysis. Thus, all collected measurements were regarded as valid. The next step was to aggregate the 15-minute data (using all data points) into hourly measurement aggregates using Python's data analysis library [26] and to match the data with the clinical data for each subject, considering also their demographic characteristics and their medical history.

### Ethics Approval

This study was approved by the institutional review board of the Sheba Medical Center, Israel (MOH_2020-07-12_009133).

## Results

A total of 410 patients, fulfilling the preliminary definition by the attending physicians as being at a high risk to deteriorate during the first 72 hours after admission, were initially recruited. The median patient age was 71 (IQR 62-78) years. Of the recruited 410 patients, 217 had undergone more than 200 measurement sessions using the wearable monitors during their hospitalization (average monitoring time 48 hours, range 25-131 hours) and thus were included in the final analysis. In total, 13 parameters were collected within each measurement session, resulting in approximately 3700 measurements per day per for each of the 217 patients included in the final analysis. When considering at least 2 days of the monitoring period, the number of data points collected during the study crossed 2,000,000 altogether. Of the 217 patients, 130 (59.9%) were male. Demographic details of the participants (upon admission) are provided in Table 1.

Baseline measurements were not significantly different between patients who deteriorated and those who did not. Actual clinical deterioration was detected by the medical staff in 24 of the 217 (11.1%) patients (Table 2).

**Figure 1:**
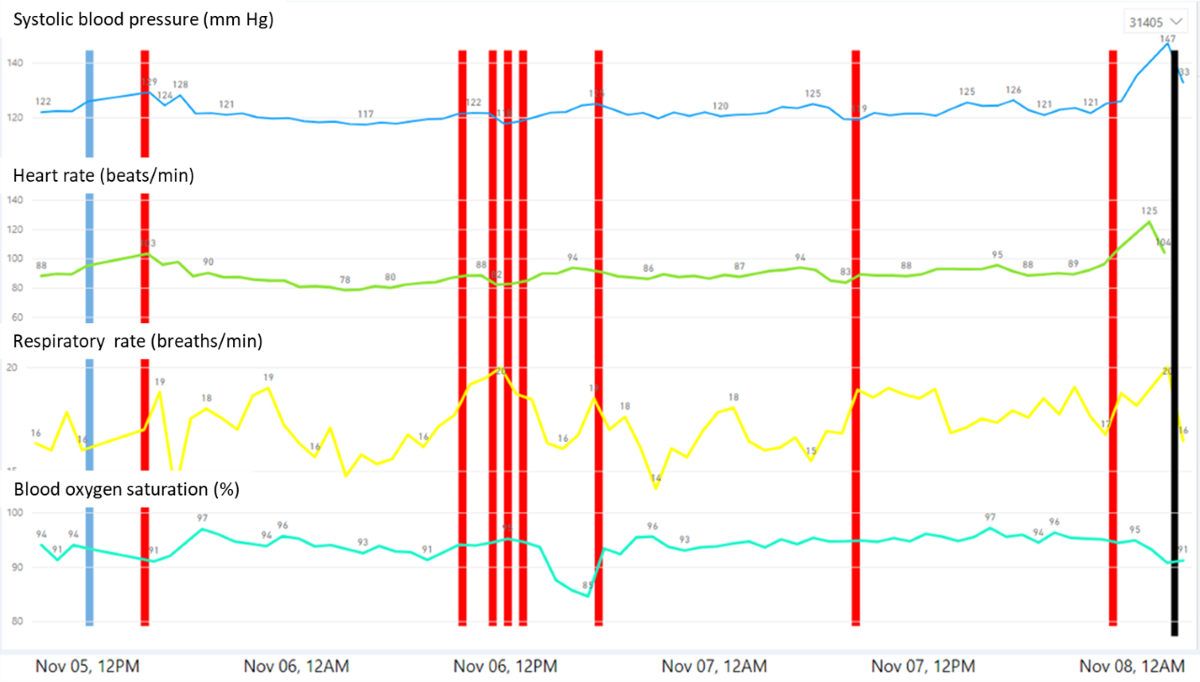
Trends of continuous data gathered by the monitoring platform. Sample of the monitoring data from a single patient, showing systolic blood pressure (mm Hg), heart rate (beats/min), respiratory rate (breaths/min), blood oxygen saturation (%), and markings of warnings and prediction. The black line indicates the time of actual clinical detection of deterioration by the medical staff. Red lines indicate times at which high-risk warnings were provided by the platform using the National Early Warning Score.

When analyzing the frequent data collected by the wearable monitors, the NEWS method provided an early warning in 16 of the 24 (67%) patients who deteriorated at 29 hours on average before actual deterioration was detected by the medical staff (an example from one patient is shown in Figure 1). The ABCNO criteria were met in 18 of the 24 (75%) patients at 38 hours on average before actual deterioration was detected by the medical staff. Early warning based on the “wish list” criteria was detected in all 24 patients at 40 hours on average before it was detected by the medical staff.

In total, 193 patients did not experience clinical deterioration during the index hospitalization. However, NEWS provided early warning alerts in 150 of the 193 (77.7%) patients, ABCNO criteria were met in 162 of the 193 (83.9%) patients, and when following the “wish list” criteria, all 193 patients who did not deteriorate had early warning alerts.

When measuring the sensitivity and specificity of the methods applied, NEWS revealed a sensitivity of 67% and specificity of 22%; ABCNO score, 75% and 16%; and the “wish list” criteria, 100% and 0%, respectively (Table 3).

**Table 1:** Demographic data of study participants with >200 measurements upon admission (N=217).

Characteristics		No deterioration (n=193)	Deteriorated (n=24)	*P* value	
Age (years), mean (SD)		70.3 (15.2)	71.8 (13.1)	.65	
Sex (male/female), n/n		115/79	16/8	.52	
BMI (kg/m^2^), mean (SD)		27.1 (5.4)	24.8 (6.2)	.05	
**Ethnicity, n**					.82
	Ashkenazy	83	11		
	Sephardi	104	13		
	Arabic	6	0		
	Other	1	0		
Blood oxygen saturation (%), mean (SD)		92.9 (10.9)	95.5 (2.2)	.30	
Respiratory rate (breaths/min), mean (SD)		17.7 (3.5)	17.2 (2.2)	.50	
Temperature (°C), mean (SD)		37.3 (0.6)	37.2 (0.6)	.31	
Heart rate (beats/min), mean (SD)		80.9 (17.7)	79.9 (17.4)	.80	
Systolic blood pressure (mm Hg), mean (SD)		129.3 (24.4)	131.0 (25.1)	.75	
Diastolic blood pressure (mm Hg), mean (SD)		68.7 (14.0)	72.2 (14.3)	.24	
Pulse pressure (mm Hg), mean (SD)		60.6 (20.9)	58.8 (24.3)	.69	
Mean arterial pressure (mm Hg), mean (SD)		88.9 (15.2)	91.8 (14.7)	.37	
Stroke volume (mL), mean (SD)		72.0 (13.6)	74.7 (17.0)	.37	
Cardiac output (L/min), mean (SD)		5.7 (0.9)	5.8 (0.9)	.39	
Cardiac index (L/min/m^2^), mean (SD)		3.1 (0.6)	3.1 (0.9)	.49	
Systemic vascular resistance (dynes•s/cm^5^), mean (SD)		1283.0 (256.1)	1305.3 (319.9)	.70	
**Background diagnosis, n (%)**					
	Ischemic heart disease	61 (31.6)	11 (46)	.17	
	Hypertension	119 (73)	17 (71)	.50	
	Congestive heart failure	21 (10.9)	4 (17)	.49	
	Diabetes mellitus	77 (39.9)	9 (38)	>.99	
	Obesity	21 (10.9)	0 (0)	.14	
	Valve disease	15 (7.8)	2 (8)	>.99	
	Chronic obstructive pulmonary disease	38 (19.7)	2 (8)	.26	
	Asthma	12 (6.2)	2 (8)	.66	
	Cerebrovascular accident	26 (13.5)	3 (13)	>.99	
	Chronic kidney disease	48 (24.9)	6 (25)	>.99	
	Epilepsy	2 (1.0)	0 (0)	>.99	
	Previous surgery	107 (55.4)	12 (50)	.67	
	Arrhythmia	60 (31.1)	8 (33)	.82	
	Anemia	27 (14.0)	3 (13)	>.99	
	Active malignancy	40 (20.7)	6 (25)	.60	
	Past malignancy	11 (5.7)	3 (13)	.19	
	Thyroid	27 (14.0)	4 (17)	.76	
	Pacemaker	13 (6.7)	1 (4)	>.99	
	Depression	8 (4.1)	0 (0)	.60	
	Bronchiectasis or cystic fibrosis	2 (1.0)	2 (8)	>.99	
	COVID-19	2 (1.0)	1 (4)	.30	
Length of stay (days), mean (SD)		2.2 (2.3)	2.4 (0.8)	.61	

**Table 2:** Comparison of different tools for early detection of patient deterioration (relating to first-time alerts only).

Tools	Patients in whom an early risk alert was generated by the scoring system, n (%)		Time of detection prior to actual clinical deterioration (hours), n
	No deterioration (n=193)	Deteriorated (n=24)	
National Early Warning Score	150 (77.7)	16 (67)	29
Airway, Breathing, Circulation, Neurology, and Other score^a^	162 (83.9)	18 (75)	38
The clinical definition of deterioration by local medical staff (“wish list”)	193 (100)	24 (100)	40

^a^A locally implemented version of the Airway, Breathing, Circulation, Disability, Exposure criteria for identification of patients' deterioration, as described in Multimedia Appendix 3.

**Table 3:** Comparison of the sensitivity and specificity of different tools of early detection of deterioration in patients with >200 measurements (N=217).

	Sensitivity, %	Specificity, %
National Early Warning Score	67	22
Airway, Breathing, Circulation, Neurology, and Other score^a^	75	16
The clinical definition of deterioration by local medical staff (“wish list”)	100	0

^a^A locally implemented version of the Airway, Breathing, Circulation, Disability, Exposure criteria for identification of patients' deterioration, as described in Multimedia Appendix 3.

## Discussion

### Principal Findings

In this study, we have assessed an automated frequent RPM platform with several integrated EWS systems. Alerts were provided many hours before patients have clinically deteriorated; however, similar alerts were also provided for patients who did not deteriorate, questioning the suitability of these EWS systems when frequent monitoring is available. EWS systems are used by health care providers to identify the early signs of clinical deterioration and initiate prompt intervention and management, including nursing staff attention, notifying the clinicians, or activating a rapid response team [27]. A numeric value is assigned to several physiologic parameters, and a composite score is derived and used to identify a patient at risk of deterioration. Most are based on an aggregate weighted system in which the elements are assigned different points for the degree of physiological abnormalities, such as those presented in Multimedia Appendices 2-4. Previous observational studies have suggested that patients often show signs of clinical deterioration up to 24 hours before a serious medical event necessitating an intervention [28]. Delays in care or insufficient treatment of patients on general hospital wards may result in increased admissions to the ICU, cardiac arrest, increased length of hospital stay, or death [28]. The purpose of the EWS scores is to ensure timely and appropriate management of deteriorating patients in general hospital wards. Moreover, for the complex patient population admitted in the general ward and the medical staff treating them, an early warning could be the difference between prevention and late response to decompensation. We also show that when using current early warning systems in a frequent measurement mode, the sensitivity is high, yet the specificity is low, potentially leading to provider fatigue in real-world settings. This is further emphasized when using the “wish list” definitions provided by the medical staff, which has led to an early warning in all 217 patients included in the final analysis, including the 193 patients who had no actual clinical deterioration. This clearly shows that the “wish list” criteria cannot be used for early warning. Previous studies have also shown the relatively high sensitivity of EWS systems; yet, among all patients, the specificity was low [29]. Moreover, in many cases, they provide too many alerts leading to alert fatigue [30].

These results might lead to claims suggesting that clinical judgment is more effective than any EWS system, highlighting the importance of holistic patient care and good clinical judgment. However, it seems that by further improving these EWS systems, sensitivity could be kept high, while specificity would be higher. This was not achieved yet, but preliminary data from various studies implementing big data analysis of multiple physiologic parameters collected automatically and frequently already show promise in early detection of clinically significant changes, and this could eventually result in the desired combination for future EWSs [24,31].

### Limitations

Though a limitation of this study is that the health care providers were not using the RPM system in real time and did not react to the warnings it provided, it is expected that in a real-world scenario, once an early warning is provided, the medical staff will intervene and provide the relevant medical support, thus changing the clinical course of the patients from that moment. Importantly, we do not know whether the alerts provided by the different early warning tools resolved spontaneously or whether patients received medical treatment coincidentally at the same time, leading to patient improvement and the resolution of the alert.

At a more practical level, we show that using an RPM system with frequent measurements is feasible in the acute care setting within the general ward. Though 410 patients were recruited, continuous monitoring was achieved properly in only 217 patients (more than 200 measurement sessions) owing to mis-attachment of the wearable monitoring devices. We assume that the reason for that was the blinding of the medical staff from access to the real-time data. Though upon attaching the monitoring devices, the research team ensured that the sensors were well attached and transmitted the data properly, from that moment on, there was no real-time and continuous indication on the quality of the signal. We did see substantial improvement with time, showing that a positive learning curve was rapidly reached and that the devices are simple to use. Moreover, in a real-time scenario, where the medical staff will rely on such a wearable monitoring system, they will immediately receive a notification of an improper signal and will reattach the sensor.

In terms of efficiency, once connected, the data were seamlessly and automatically transferred into the data collection repository and, in parallel, could have been presented on the monitoring screens of the department. This part was not available to the medical staff as they were blinded to real-time monitoring data. EWS compliance is often found to be poor for several reasons, including misinterpretation or incorrect calculation of the scores and poor communications [32]. However, this becomes irrelevant when using an automatically generated and transmitted EWS score.

Further development and future studies are needed to provide an advanced EWS tool that would have higher sensitivity and specificity, making it a better-suited tool in real-world settings, focusing on presymptomatic warnings of potential patient deterioration, to be used as a preventive measure and as a medical decision support tool in both the outpatient and in-hospital settings. The combination of frequently collected multiple physiological parameters, an advanced algorithm, and timely alerts could potentially provide medical staff peace of mind, knowing that they are called only when there is an imminent threat of clinical importance. Moreover, improved prediction of deterioration would have vast positive outcomes when considering the low availability of telemetry beds in hospitals.

Another limitation of the study is that we did not have continuous measurements for all patients during the whole monitoring period. Nonetheless, the data set is much larger than what usually is collected within the general wards, and it still provides important insights. This should be further optimized in future studies on this subject.

### Conclusions

To conclude, frequent RPM allows for early detection of physiological changes with potential clinical significance. The integration of an EWS system may provide another layer of clinical awareness, serving as an important decision support tool for early medical intervention. Current scoring systems have high sensitivity but low specificity and warrant further development when combined with frequent multiparameter monitoring. The frequency of measurements alone, though providing a better understanding of trajectories of various vital signs, is not enough to provide an improved EWS score, and in practice, this might be translated into a high rate of alarms, complicating its hospital implementation.

Future systems, which would rely on frequent collection and calculation of the EWS score, could provide better sensitivity and specificity and should be better adjusted to provide tailored scores for the prevention of different medical conditions.

## Appendix

Multimedia Appendix 1 CONSORT Patient and Analysis Flow.

Multimedia Appendix 2 National Early Warning Score (NEWS).

Multimedia Appendix 3 Medical Emergency Team activation criteria.

Multimedia Appendix 4 Deterioration criteria are defined by the clinical teams of the general wards assuming continuous monitoring available.

## Abbreviations

ABCNO: Airway, Breathing, Circulation, Neurology, and Other
AI: artificial intelligence
EWS: early warning score
ICU: intensive care unit
NEWS: National Early Warning Score
RPM: remote patient monitoring
