# Supplementary figures and images for: Assessing the Usability of a Novel Wearable Remote Patient Monitoring Device for the Early Detection of In-Hospital Patient Deterioration: Observational Study
Source: JMIR Form Res. 2022 Jun 9;6(6):e36066. doi: 10.2196/36066 (PMC9227660; doi:10.2196/36066)

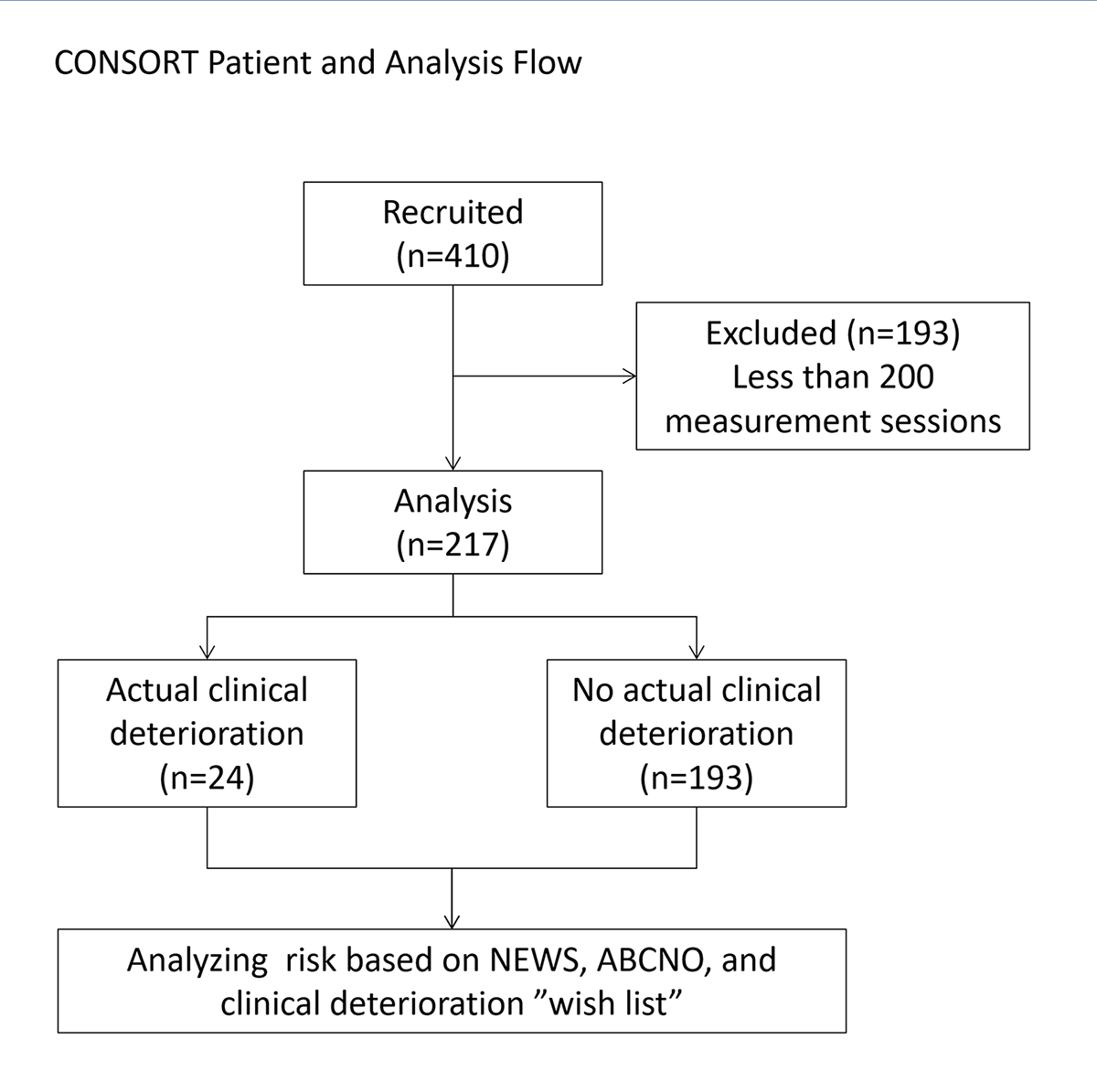

Supplement: Multimedia Appendix 1 [file formative_v6i6e36066_app1.png]
