# Supplementary material for: Assessing the Usability of a Novel Wearable Remote Patient Monitoring Device for the Early Detection of In-Hospital Patient Deterioration: Observational Study
Source: JMIR Form Res. 2022 Jun 9;6(6):e36066. doi: 10.2196/36066 (PMC9227660; doi:10.2196/36066)

**National Early Warning Score (NEWS)**. This tool was developed by the Royal College of Physicians [16-19] to improve the detection and response to clinical deterioration in adult patients, thus helping with patient safety and patient outcomes. Respiratory rate (BPM) – breathes per minute. Systolic blood pressure – values are in mmHg units. Heart rate (BPM) – beats per minute. ACVPU: alert, confusion, voice, pain, unresponsive.


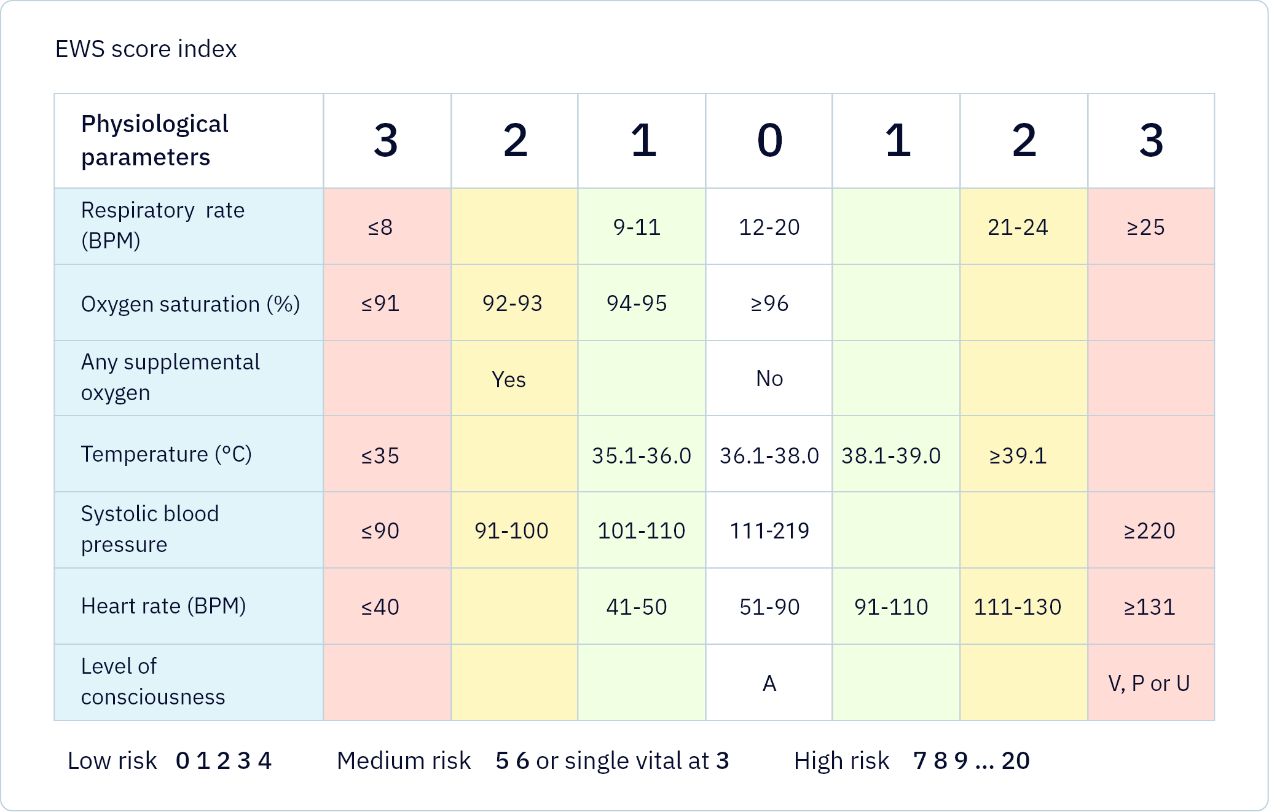

Supplement: Multimedia Appendix 2 [file formative_v6i6e36066_app2.docx]
