# Supplementary material for: Assessing the Usability of a Novel Wearable Remote Patient Monitoring Device for the Early Detection of In-Hospital Patient Deterioration: Observational Study
Source: JMIR Form Res. 2022 Jun 9;6(6):e36066. doi: 10.2196/36066 (PMC9227660; doi:10.2196/36066)
Supplement: Multimedia Appendix 3 [file formative_v6i6e36066_app3.docx]

**Medical Emergency Team activation criteria, also known as the ABCNO criteria, as implemented at the study site** (the ABCNO (airway; breathing; circulation; neurology and other) is a version of the ABCDE (airway; breathing; circulation; disability and exposure) risk score for the deterioration that is routinely used in our facility) (19). The protocol does not outline the percentage or flow of oxygen delivery.

| Criterion | Description |
| --- | --- |
| Airway | Respiratory distress; concern regarding airway |
| Breathing | Respiratory rate > 30/minute or < 8/min; Oxygenation < 90% on oxygen |
| Circulation | Systolic blood pressure < 80 mmHg or > 200 mmHg; Heart rate > 130 beats/minute or < 40 beats/minute |
| Neurology | Decrease in the conscious state (based on the Glasgow Coma Scale); Seizures |
| Other | Concern about the patient |
