# Supplementary material for: Assessing the Usability of a Novel Wearable Remote Patient Monitoring Device for the Early Detection of In-Hospital Patient Deterioration: Observational Study
Source: JMIR Form Res. 2022 Jun 9;6(6):e36066. doi: 10.2196/36066 (PMC9227660; doi:10.2196/36066)
Supplement: Multimedia Appendix 4 [file formative_v6i6e36066_app4.docx]

**Deterioration criteria are defined by the clinical teams of the general wards assuming continuous monitoring available.** Defined hereafter as the "wish list" that would provide them with the ideal early warning of risk to patients. SPO2 – blood oxygen saturation.

| Criterion | Description |
| --- | --- |
| Respiratory rate | 20% increase from admission; respiratory rate > 20/minute or < 8/min while awake |
| SPO2 | Oxygenation < 93% (with or without oxygen supplement) |
| Heart rate | 15% increase in heart rate from admission; Heart rate > 150 beats/minute or < 50 beats/minute |
| Systolic blood pressure | 20% increase or decrease from admission; systolic blood pressure < 85 mmHg or > 150 mmHg |
| Diastolic blood pressure | 10% increase or decrease from admission; diastolic blood pressure < 50 mmHg or > 100 mmHg |
| Temperature | Increase or decrease of 1 °C from admission; > 37.8 °C |
